# Cinnamon essential oil and its emulsion as efficient antibiofilm agents to combat *Acinetobacter baumannii*

**DOI:** 10.3389/fmicb.2022.989667

**Published:** 2022-10-10

**Authors:** Tea Ganić, Stefana Vuletić, Biljana Nikolić, Magdalena Stevanović, Maja Kuzmanović, Dušan Kekić, Saša Đurović, Stefana Cvetković, Dragana Mitić-Ćulafić

**Affiliations:** ^1^Faculty of Biology, University of Belgrade, Belgrade, Serbia; ^2^Group for Biomedical Engineering and Nanobiotechnology, Institute of Technical Sciences of SASA, Belgrade, Serbia; ^3^Faculty of Medicine, Institute of Microbiology and Immunology, University of Belgrade, Belgrade, Serbia; ^4^Institute of General and Physical Chemistry, Belgrade, Serbia

**Keywords:** *Acinetobacter*, antibacterial, biofilm, cinnamon, cytotoxicity, emulsion, essential oil, genotoxicity

## Abstract

*Acinetobacter baumannii* is an emerging nosocomial pathogen resistant to a wide spectrum of antibiotics, with great potential to form a biofilm, which further aggravates treatment of infections caused by it. Therefore, searching for new potent agents that are efficient against *A. baumannii* seems to be a necessity. One of them, which has already been proven to possess a wide spectrum of biological activities, including antimicrobial effect, is cinnamon essential oil. Still, further increase of antibacterial efficacy and improvement of bioavailability of cinnamon oil is possible by emulsification process. The aim of this study was comparative analysis of cinnamon essential oil and its emulsion against biofilm forming *A. baumannii* clinical isolates. Furthermore, the investigation of toxicological aspects of possible applications of essential oil and emulsion was done as well. Gas chromatography–mass spectrometry of essential oil indicated *trans*-cinnamaldehyde as the most abundant component. The cinnamon emulsion was synthesized from cinnamon essential oil by combining modified low- and high- energy methods. Synthesized emulsion was characterized with Fourier-transform infrared spectroscopy and photon correlation spectroscopy. Both substances exhibited significant antibacterial (minimal inhibitory concentrations in the range 0.125–0.5 mg/ml) and antibiofilm effects (inhibitions of formation and reduction of pre-formed biofilm were 47–81 and 30–62%, respectively). Compared to essential oil, the efficacy of emulsion was even stronger considering the small share of pure oil (20%) in the emulsion. The result of biofilm eradication assay was confirmed by scanning electron microscopy. Even though the cytotoxicity was high especially for the emulsion, genotoxicity was not determined. In conclusion, strong antibacterial/antibiofilm effect against *A. baumannii* of the cinnamon essential oil and the fact that emulsification even potentiated the activity, seems to be of great significance. Observed cytotoxicity implicated that further analysis is needed in order to clearly determine active principles being responsible for obtained antibacterial/antibiofilm and cytotoxic properties.

## Introduction

Essential oils (EOs) are the plant’s secondary metabolites that have been used for thousands of years as medicines, food spices, raw materials for perfumes and other cosmetic preparations, as well as in cult rituals. They originated from aromatic plants from families like Lauraceae, Lamiaceae, Myrtaceae, Asteraceae and others. Different plant parts could be used as a source of EO, such as bark, leaves, blooms or the whole plant (Ni et al., 2021). Primarily, EO consists of volatile compounds and commonly, only a few of them are predominantly presented, with up to 70% contribution to overall chemical composition (Swamy et al., 2016; Behbahani et al., 2020). The most present chemical compounds which could be found as constituents are terpenes, terpenoids, phenylpropanoids, flavonoids, aldehydes and similar (Moghaddam and Mehdizadeh, 2017; Chaudhari et al., 2021). Besides many positive characteristics of EOs, some of their constituents could potentially be harmful to health, so careful use is needed. The disadvantage of EO utilization as a source of natural bioactive compounds is that they are not water-soluble, which makes their application difficult. Furthermore, additional problems in their applications in the pharmaceutical and cosmetic industries could arise from their extreme volatility and sensitivity to light exposure and elevated temperature (Dima and Dima, 2015). The possibility of synthesis of EOs into hydrophilic drops and constructing their emulsions could help overcome some of the listed problems. Prepared emulsion of EO provides better solubility and stability which facilitates its incorporations in gels and creams, and also, protects them from degradation (Barradas and de Holanda e Silva, 2021). The small size but the large surface area of nanoparticles contributes to facilitated membrane penetration. When integrated, the EOs’ components could be released in a controlled manner into its surroundings and the influence of its emulsion lasts longer (Barradas and de Holanda e Silva, 2021).

*Cinnamomum zeylanicum* is one of the oldest known herbal spices and folk medicine, used for approximately 4,000 years in different aspects of human lives. It was believed that cinnamon heals fevers, toothache, colds, tumors, diabetes and so on. This species belongs to the Lauraceae family and is widely spread in Sri Lanka and other Asian countries (Wong et al., 2014; Mohamed et al., 2020). *Cinnamomum zeylanicum* EO has a wide spectrum of biological activities such as antimicrobial, antifungal, antioxidant, anticancer, anti-inflammatory and antidiabetic (Ribeiro-Santos et al., 2017). As cinnamon EO possesses an antimicrobial effect its emulsion is potentially a fine way to facilitate its application and eventually improve activity. According to the literature, the antimicrobial effect of cinnamon EO has been investigated so far, but data on the antibiofilm effect are rare. Moreover, data on the antimicrobial/antibiofilm activity of cinnamon EO emulsion (EM) are particularly scarce, indicating the importance of its investigation. This is especially important in the light of the growing bacterial resistance to conventional antibiotics (Darwish and Aburjai, 2010).

One of the multidrug-resistant bacteria is *Acinetobacter baumannii*, a gram-negative, opportunistic pathogen, most common in intensive care units that could induce severe consequences, especially for patients with an immunocompromised immune system (Bamunuarachchi et al., 2021). Usually, they are associated with respiratory and urinary tract infections or even bacteremia. Recently *A. baumannii* has been proclaimed as the most critical group, resistant to different classes of antibiotics (Thirapanmethee et al., 2021). It belongs to the ESKAPE (*Enterococcus faecium*, *Staphylococcus aureus*, *Klebsiella pneumoniae*, *Acinetobacter baumannii*, *Pseudomonas aeruginosa*, *Enterobacter* species) group of pathogens and due to its potential to easily overcome antibiotic treatments, alternative strategies to combat the infections caused by it are necessary. *A. baumannii* easily survives in different surroundings, possibly thanks to its ability to form biofilm (Sarshar et al., 2021). Biofilms represent the microbial community that can be formed on different surfaces. Bacteria are coated with a matrix and protected from external impacts. Matrix, thanks to its properties, prevents antibiotic penetration and their action. Biofilm-related infections are difficult to treat, so a way to avoid these infections is the prevention of biofilm formation (Gedefie et al., 2021). However, science should search for opportunities to overcome infections that are developed due to already formed biofilms. In such situations, EM application seems to have great potential, because of the small size of nanoparticles that can diffuse more easily and deeper through a matrix to the bacterial cells and manifest antibacterial effect (Nabawy et al., 2022).

Even so nanotechnology sounds promising as a drug delivery system, which also provides more stable drugs and their controlled release in organisms, there is much to learn about the possible side effects before the use of such development (Das et al., 2021; Khan et al., 2021a). Toxicity is directly related to the components from which emulsions are synthesized, but also its structure. They are made of natural or synthetic components, oils, surfactants and cosurfactants, all used in small amounts (Khan et al., 2021b, 2022). Nevertheless, even though many of the components are proved not to be toxic when they have been applied alone, there is still the possibility that some of them could be harmful, especially if they are mixed into an emulsion. Because of the small sizes of nanodrops, bioavailability is higher so the active components are easy to adopt, which could be considered a double-edged sword: although the beneficial effects of the bioactive substances are increased, they could also easily induce some negative consequences. The intensity and electrification of the charge of nanostructures affect emulsion stability, but also its interactions with cells, including harmful ones (Wani et al., 2018; Hort et al., 2021). Furthermore, the fact is that nanoparticles could easily achieve DNA and consequently due to high bioactivity could interact with it and induce genotoxic effect (Shukla et al., 2021). Therefore, investigating emulsion safety, i.e., possible cytotoxic and genotoxic activity seems to be of crucial importance. Taking all abovementioned into consideration, this study aimed to investigate antibiofilm effect of the cinnamon EO against the emerging pathogen *A. baumannii*, as well as determine whether the emulsification of EO could increase efficacy. Furthermore, an additional aim was to provide a risk assessment of cinnamon EO and EM for possible human use, i.e., their cytotoxicity and genotoxicity were provided as well.

## Materials and methods

### Cinnamon essential oil and emulsion synthesis

**Figure 1:**
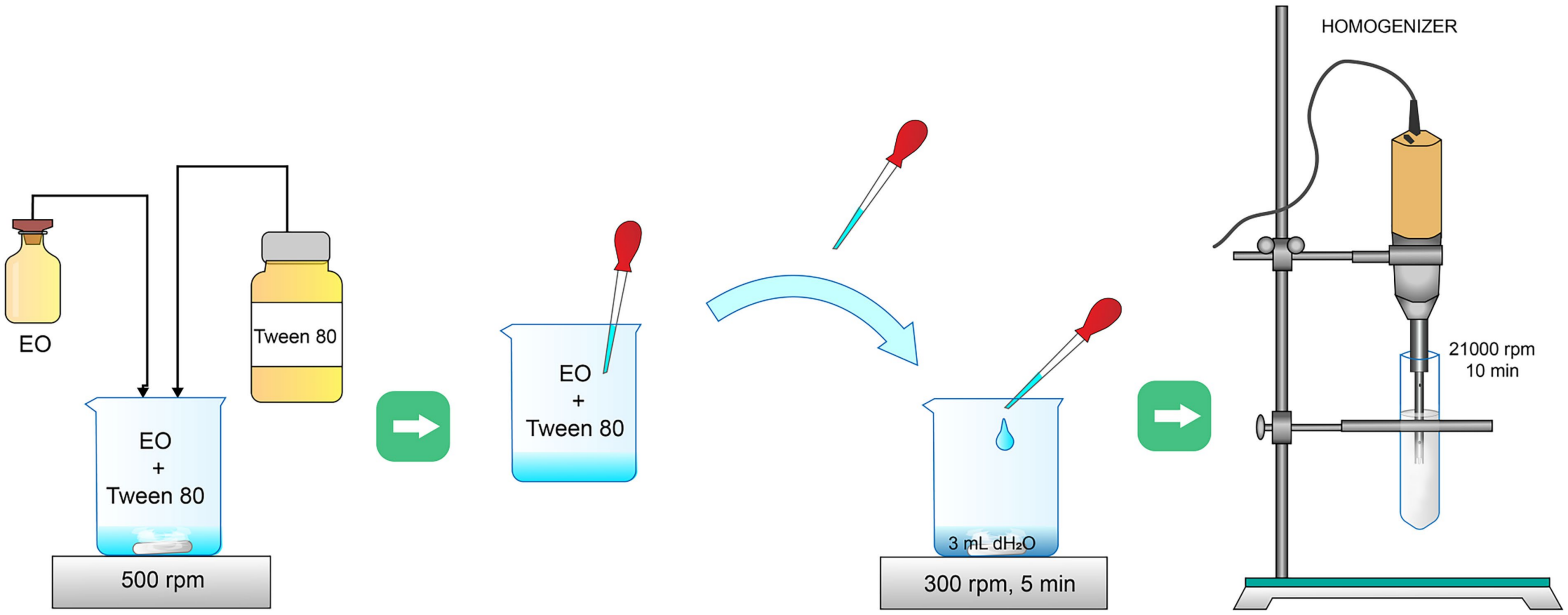
Methodology of cinnamon emulsion synthesis.

Cinnamon EO (*Cinnamomum zeylanicum* L.; P0125285), extracted from the bark, was commercially obtained (Frey + Lau GmbH, Henstedt-Ulzburg, Germany). Its EM was synthesized by combining modified low- and high-energy methods, both very often used in the literature (Gupta et al., 2017). In literature, the most commonly used low-energy method for the preparation of emulsions is the so-called emulsion inversion point method, where components are mixed in a specific order at constant temperature and constant velocity on a magnetic stirrer. In this study, cinnamon EO, Tween 80 as a surfactant, and deionized water were used to prepare the EM. Tween is a non-ionic ester, hydrophilic in nature, and suitable for the formation of an oil-in-water emulsion (Florence and Rogers, 1971). Firstly, 1 ml of surfactant Tween 80 was added to 1 ml of EO, with continuous stirring at 500 rpm by a magnetic stirrer (Figure 1). Then, the obtained oil/surfactant mixture was added drop-wise in 3 ml of deionized water during 5 min of stirring at 300 rpm on a magnetic stirrer, with the resulting solution becoming milky white. Afterward, the obtained suspension was transferred to a tube and homogenized at 21,000 rpm for 10 min. Homogenization is the high-energy method that was used to make sure that large droplets are broken into smaller sizes.

### Gas chromatography/mass spectrometry (GC/MS) analysis of essential oil

Cinnamon EO was characterized using gas trap GC/MS (Thermo Fisher, MA, United States), as described by Micić et al. (2021), in order to define its chemical composition. The sample has been dissolved in methylene chloride and used capillary column was TR WAX-MS (30 m × 0.25 mm, 0.25 μm). The initial temperature was held at 45° C for 8 min, then increased for 8.0°C/min to 230°C and held for 10 min. The gas carrier was helium 1 ml/min at split ratio of 80:1. Component identification was done comparing mass spectra and the National Institute of Standards and Technologies 08 MS database and with the spectra of available standards. Results were presented as relative percentage (%) and the final content of compounds in milligram per milliliter (mg/ml).

### Fourier-transform infrared spectroscopy

The cinnamon EM, as well as a cinnamon EO, were analyzed by Fourier transformed infrared spectroscopy (FTIR) in order to perform further physicochemical characterization of the samples, i.e., to perform quality analysis and determine the presence of specific functional groups. FTIR spectra of the samples were recorded in the range of 400–4,000 cm^−1^ using a Thermo Scientific Nicolet iS10 Spectrometer equipped with Smart iTX accessory (Thermo Fisher Scientific Inc. United States) at 4 cm^−1^ spectral resolution and 32 scans. When the sample is deposited, evaporation in the air occurred which avoided spectra saturation due to water contained in the emulsion. FTIR spectra were collected in reflection mode with a built-in diamond Attenuated Total Reflectance (ATR) sampling technique. The OMNIC Software was used for the acquisition, processing, analysis, and managing of FTIR data in a graphical environment.

### Determination of droplet/particle size

Samples of EM were analyzed without prior dilution. The average droplet/particle size (Z-ave) and polydispersity index (PdI) in the tested samples were determined by photon correlation spectroscopy (PCS) using a ZetasizerNano ZS90 (Malvern Instruments, Worcestershire, United Kingdom) equipped with He–Ne laser light with a wavelength of 633 nm. The detection of scattered light was performed at an angle of 90°. All measurements were performed at a temperature of 25 ± 0.1°C. Measurement data were analyzed with Dispersion Technology Software (DTS), and the obtained results were presented as the mean of three consecutive measurements ± S.D.

In addition, the zeta potential of EM samples was measured by Zetasizer (Zeta-sizer Nano-Z, Malvern, United Kingdom) using a 4 mW He–Ne laser (*λ* = 632.8 nm) and using the principle of electrophoretic mobility under an electric field. Zeta potential is the function of the dispersion/suspension pH. The zeta potential of the EM was studied at a pH of 6.5 by sampling 0.1 ml of the emulsion from the stock emulsion and homogenization or mixing it with distilled water. Zeta potential is an important tool for understanding the state of the particle surface and predicting its long-term stability. The electrostatic repulsion between particles depends on the value of zeta potential (Hunter, 2013).

### Bacterial strains

Bacterial isolates (Table 1) for this research were obtained from the collection of the Medical Faculty, University of Belgrade. There were four clinical isolates of the *Acinetobacter baumannii*, being previously identified and characterized as multidrug-resistant (MDR) strains (Lukovic et al., 2020). Among used isolates, GN249 and GN321 were invasive. In addition, referent ATCC 19606 strain was used. Bacterial strains were cultivated in Luria Broth (LB, Sigma Aldrich, Steinheim, Germany) medium at 37°C and stored on Luria Agar (LA, Sigma Aldrich, Steinheim, Germany) medium at the 4°C.

**Table 1:** *Acinetobacter baumannii* strains.

Strain	Origin	
ATCC 19606	Collection	Institute of Microbiology and Immunology, Faculty of Medicine, University of Belgrade
GN 249	Central venous catheter	
GN 321	Blood	
GN 1028	Tracheal aspirate	
GN 1031	Swab wound		

### Microdilution assay

Defining of minimal inhibitory concentration (MIC) was performed with the microdilution-broth assay, described by Đukanović et al. (2020). The bacterial suspension was prepared to be 1 × 10^4^cell/well. The assay was done in 96 well plates and concentrations of EO and EM were in a range from 0.031 to 4 mg/ml. Serial of two-fold dilution was done in Mueller Hinton Broth (MHB, Titan Biotech Limited, Rajasthan, India), directly in the plates. Positive control was antibiotic colistin (Sigma Aldrich, Steinheim, Germany), tested in the same concentration range. EO was dissolved in dimethylsulfoxide (DMSO, Serva Electrophoresis GmbH, Heidelberg, Germany) and EM in Tween 80 (Fisher Scientific, Waltham, MA, United States), and each solvent was used as a solvent control for up to 2.5 and 0.2% of well content, respectively. After 24 h incubation at 37°C, cell viability was measured at the absorbance of 600 nm, on a micro-plate reader (Multiskan, FC, Thermo Scientific, Shanghai, China) and MIC concentration was confirmed with resazurin staining. Incubation with added resazurin (0.675 mg/ml, Serva Electrophoresis GmbH, Heidelberg, Germany, Cas no:62758-13-8) was 3 h long at 37°C, and MIC values were determined as the lowest concentrations that showed no visible color change. The assay was performed with EO and EM, as well as with colistin, used as a positive control.

### Biofilm assay

#### Antibiofilm assay

Antibiofilm assay was done in 96 well plates, with treatment concentrations range 1/64 MIC–MIC. Biofilm biomass was quantified with Crystal Violet (CV, *Lach-Ner,s.r.o,* Brno, Czech Republic) staining, as previously described by Đukanović et al. (2022) with slight changes. Briefly, the medium prepared for the experiment was TSB (Tryptic Soy Broth, Titan Biotech Limited, Rajasthan, India) with added 0.5% glucose. Bacterial inoculum was prepared at a final concentration of 1 × 10^6^ cell/well, and treated with EO and EM in the previously mentioned concentration range. The plates were incubated for 24 h at 37°C, and after that time the biofilm biomass was stained with CV. In brief, wells were rinsed with water, carefully, in order not to disrupt the biofilm. An 0.1% CV solution was added and incubated for 20 min at room temperature. Plates were rinsed with water and the rest of the colored biomass biofilm was dissolved in ethanol. Biofilm biomass was measured on a Micro-Plate reader (Multiskan, FC, Thermo Scientific, Shanghai, China) at the optical density (OD) 570 nm. Biofilm biomass was calculated as a percentage of biofilm biomass of control without the treatment and was calculated according to the formula:


$$\%\mathrm{Biofilm biomass}=100\times \frac{{OD}_{570\;\left(\mathrm{Sample}\right)}}{{OD}_{570\left(\mathrm{Control}\right)}}\text{.}$$

The experiment was done three times and each one in hexaplicates per plate.

#### Biofilm dispersal assay

Biofilm dispersal assay was also done in 96 well plates, but the treatment concentration range was from ½ MIC to 4 MIC and treatment was performed on pre-formed biofilms. The bacterial suspension was prepared the same as previously, 1 × 10^6^ cell/well in TSB medium with added 0.5% glucose. After 24 h incubation at 37°C, formed biofilm was treated with EO and EM for an additional 24 h at 37°C. On the third day biofilms were rinsed and stained with CV, and biofilm biomass was quantified exactly as it was mentioned above. An experiment was done three times in hexaplicates. Furthermore, the effect of cinnamon EO and EM on *A. baumannii* biofilm was monitored using Scanning Electron Microscopy (SEM). Bacterial cultures were diluted up to 10^6^ cell/well and biofilms were cultivated on cover glasses, each placed in well with TSB media with added 0.5% glucose. The plates were incubated on 37° C for 24 h. The next day, media was changed with the fresh, added the treatment in 4MIC concentrations and left for another 24 h in incubation on 37° C. On the third day, cover glasses were rinsed with the PBS (Dulbeccos Phosphate Buffered Saline, Sigma Aldrich, Steinheim, Germany) and left to dry out in the air. Fixed cover glasses were coated with approximate thickness of gold 15–20 nm, using a BALTEC-SCD-005 sputter coating instrument. Monitoring biofilms on SEM, model JOEL JSM-6610LV, equipped by an EDS, an Oxford X-Max energy dispersive spectrometer, was done as it was described by Đukanović et al. (2020).

### Cell line

The cell line chosen for the experiment was MRC-5 (ECACC 84101801) i.e., normal lung fetal fibroblasts. They were kindly provided by Sergej Tomić, PhD, Institute for the Application of Nuclear Energy (INEP). The cells were grown in Dulbecco Modified Eagle’s Medium (DMEM, Thermo Fisher Scientific, MA, United States) with 10% Fetal Bovine Serum (FBS, Sigma Aldrich, Steinheim, Germany), 2 mM L-glutamine (Serva Electrophoresis GmbH, Heidelberg, Germany) and 100 U/ml penicillin/streptomycin (Sigma Aldrich, Steinheim, Germany) at 37°C, with 5% CO_2_ in the atmosphere. When the confluent cell growth reached, about 90% of the surface of the flask, they were sub-cultured with 0.1% trypsin solution (Sigma Aldrich, St. Louis, MO, United States).

### Cytotoxicity assay

Cytotoxicity of EO and EM was performed using a colorimetric method MTT (3-(4,5-dimethylthiazol-2-yl)-2,5-diphenyltetrazolium bromide, Sigma Aldrich, Heidelberg, Germany, Cas 298-93-1) assay, as previously described by Cvetković et al. (2020). For the purposes of this experiment MRC-5 cell line was trypsinised with 0.1% trypsin solution and seeded in 96 well plate with 2 × 10^4^ cell/well. Plate with cells was incubated at 37°C for 24 h in order to form confluent growth in each well. Tested concentrations of EO were in a range from 0.015 mg/ml to 1 mg/ml and for EM were from 0.007 to 0.5 mg/ml. During the treatments, cells were incubated for 24 h, at 5% CO_2_, in a humidified atmosphere. The next day medium from the wells was carefully extracted and replaced with MTT solution (final concentration 0.5 mg/ml). At the end of the incubation with MTT, the medium was removed, and the formazan crystals were dissolved in DMSO. Cell viability was measured using micro-plate reader (Multiskan, FC, Thermo Scientific, Shanghai, China) at the 570 nm wave length. The absorbance of treated cells was compared with the untreated control, i.e., the solvents was used as a control (0.5% DMSO for EO and 0.05% Tween 80 for EM). The percentage of viability was calculated with the equation:


$$\%\mathrm{Cell viability}=100\;x\;\frac{{OD}_{570\;\left(\mathrm{Sample}\right)}}{{OD}_{570\left(\mathrm{Control}\right)}}\text{.}$$

### Comet assay

The alkaline comet test was applied as previously described by Cvetković et al. (2022) with slight modifications, to determine DNA damage. MRC-5 cell line was seeded in 12-well plates with 3x10^6^cell/well and left in incubator at 37° C and 5% CO_2_ humified air for 24 h. After that time, the cells were treated with EO and EM in concentrations ranging from 0.0156 to 0.125 mg/ml and from 0.001 to 0.031 mg/ml, respectively. Treated cells were then incubated for 24 h under the same conditions. As a positive control hydrogen-peroxide (0.1%, *v*/*v*) was used. The comet assay was performed as follows: cell suspension (30 μl) was mixed with 70 μl of 1% LMP (low melting point, Bio Rad Laboratories, Hercules, CA, United States) and added on previously coated microscopic slides with the 1% NMP (normal melting point, Serva Electrophoresis GmbH, Heidelberg, Germany) agarose solution. The slides were lysed (NaCl 146.6 g, EDTA 37.2 g, Tris1.21 g, dH_2_O 1,000 ml, 1% Triton-X-100; pH 10) and left for 1 h at 4°C and afterward moved and kept at the electrophoresis bath with alkaline buffer (10 M NaOH 30 ml, 0.2 M EDTA 5 ml, dH_2_O 965 ml; pH13) for 20 min at 4°C. After that time, electrophoresis lasted 20 min, at 0.75 V/cm and 300 mA. In the end, microscopic slides were put in a neutralization buffer (Tris 48.44 g, dH_2_O 1,000 ml; pH7.5) for 20 min. Cells nuclei were scored by use of a fluorescent microscope (Leica, DMLS, Vienna, Austria) and computer software IV Comet (Perceptive Instruments, Edmunds, United Kingdom). Fifty nuclei per treatment were analyzed based on tail intensity as a parameter for DNA damage.

### Statistical analysis

Biofilm and MTT assays results were processed in GraphPad Prism 6.01. Software. The applied test was One-Way ANOVA, Dunnet’s *post hoc* test, used in order to present mean values with standard deviations. For Comet assay results were analyzed in Statistica 7.01 Software (StatSoft, Inc.). After determining if data were normally distributed using the Kolmogorov–Smirnov test results were further analyzed with a nonparametric Mann–Whitney *U* test. Significant levels were adjusted to be *p* < 0.001 for comet assay, and *p* < 0.05 for all the rest.

## Results

### GC/MS analysis of cinnamon essential oil

Obtained results in this study provided a complete chemical profile of the EO. In Table 2 identified chemical compounds are presented. The most abundant chemical compound is *trans*-cinnamaldehyde with a ratio of 61.9%. *Trans*-cinnamaldehyde belongs to the phenylpropanoid group of compounds with an aromatic ring and three-carbon propene tail. The following components in EO were cinnamyl acetate (7.56%), eugenol (5.96%), anethole (4.43%) and *trans*-caryophyllene (4.04%), in a noticeably smaller amount.

**Table 2:** Gas chromatography–mass spectrometry analysis of cinnamon essential oil.

Compound	%	Content (mg/g EO)
α-Pinene	0.79	5.84 ± 0.09
β-Pinene	0.15	0.28 ± 0.03
α-Terpinolene	0.04	–
Limonene	1.57	12.29 ± 0.15
Eucalyptol	2.72	22.24 ± 0.20
γ-Terpinolene	0.04	0.08 ± 0.00
p-Cymene	2.31	12.00 ± 0.11
cis-Linalool oxide	0.01	–
Cubebene	0.01	–
trans-Linalool oxide	0.01	–
α-Copaene	0.12	–
Benzaldehyde	0.3	–
Linalool	3.35	40.40 ± 0.26
Longifolene	0.06	–
1-Terpinoel	0.05	0.31 ± 0.01
**trans-Caryophyllene**	**4.04**	**25.54 ± 0.22**
β-Terpineol	0.1	0.69 ± 0.02
Humulene	0.15	–
Neodihydrocarveol	0.05	–
α-Terpineol	1.69	19.85 ± 0.11
Benzyl acetate	0.01	–
β-Cadinene	0.01	–
1,3-Hexadienylbenzene	0.02	–
2,4-Hexadienylbenzene	0.03	–
**Anethole**	**4.43**	**21.76 ± 0.18**
Benzyl alcohol	0.11	–
cis-Cinnamaldehyde	0.29	–
3-Phenylpropyl acetate	0.03	–
Anisaldehyde	0.02	–
Caryophyllene oxide	0.14	0.38 ± 0.01
α-Methylcinnamaldehyde	0.04	–
**Trans-Cinnamaldehyde**	**61.9**	–
**Cinnamyl acetate**	**7.56**	–
**Eugenol**	**5.96**	**38.72 ± 0.22**
Cinnamyl alcohol	0.26	–
α-Methoxycinnamaldehyde	0.75	–
Coumarin	0.04	–
Benzyl benzoate	0.83	–

The bold values represent the most abundant components in the cinnamon essential oil.

### Physico-chemical characterization of emulsion

**Figure 2:**
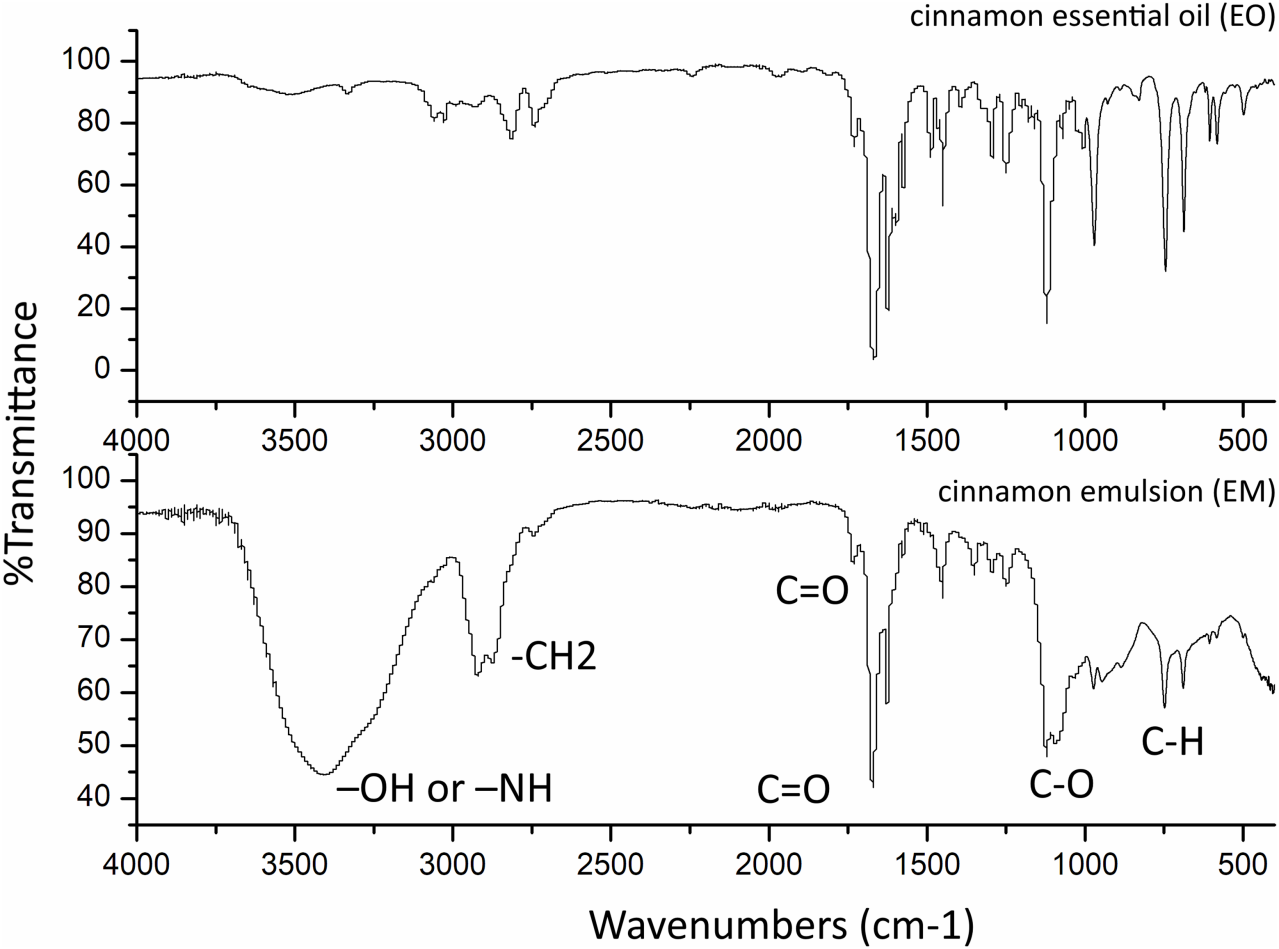
Fourier-transform infrared spectroscopy spectra of the cinnamon essential oil and as prepared cinnamon emulsion.

FTIR spectra (Figure 2) of the EO and EM, were recorded in the range of 400–4,000 cm^−1^ using a Thermo Scientific Nicolet iS10 Spectrometer. Analysis of the samples EO and EM revealed the presence of the characteristic carbonyl stretching absorptions as one of the strongest bands (1,730–1,690 cm^−1^) corresponding to typical trans-cinnamaldehyde (Boughendjioua et al., 2017) which is also in agreement with the GC/MS study where this compound was determined to be the most abundant. However, unlike EO, the spectrum of EM exhibited a broad absorption band at 3407.96 cm^−1^ which can be assigned to –OH or –NH vibrations. From the literature, it is known that Tween 80, which is used as a surfactant in the synthesis of EM, confines a significant number of hydroxyl groups, which exhibit an intense broad stretching vibration in that region (Roy Choudhury et al., 2013). From the literature, in addition it is known that Tween 80 besides hydroxyl stretching vibrations also has asymmetric and symmetric stretching bands of –CH2, and a stretching band of the C=O ester group (Ren et al., 2012). At the FTIR spectrum of EM, these asymmetric and symmetric stretching bands of –CH2 are at 2,921 and 2,893 cm^–1,^ respectively, and a band of the C=O is at 1,732 cm^−1^. The bands at 1,050–1,200 cm^−1^ belong to C–O stretch vibration while bands at 680–860 cm^–1^ belong to aromatic C–H bending.

**Figure 3:**
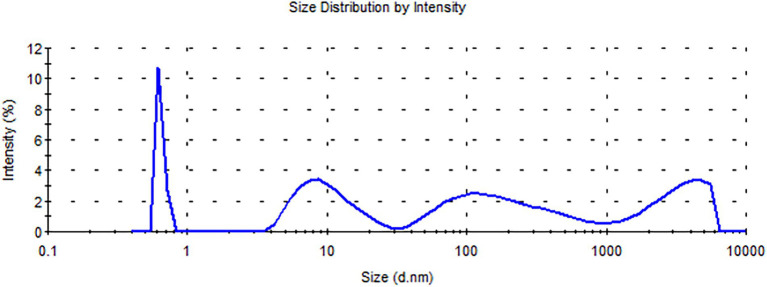
Droplet/particle distribution by intensity curve for the cinnamon.

Obtained results of particle size measurements by PCS reveal multimodal droplet/particle distribution in the case of the cinnamon’s emulsion. The multimodal particle size distribution is probably caused by the mixing of multiple particles and their agglomeration. Agglomeration can be related to a phenomenon of interparticle adhesion and flocculation (Endres et al., 2021). However, in this case, there is an even greater possibility that the formation of hydrates has occurred near the water–oil interface which in turn caused the creation of oil-droplet chains (Guo et al., 2020). Size distribution ranged from less than 1 nm to approximately 6,000 nm, which is presented in Figure 3.

Zeta-potential determinations were usually conducted for examining the adsorption-induced wettability alteration and the stability of oil/water emulsions (Mandal and Bera, 2015). As already mentioned above, the EM was prepared using only Tween 80 as a surfactant which is a non-ionic ester, hydrophilic in nature. From the literature, stabilization of emulsion by non-ionic surfactants is due to steric repulsion while in case of ionic surfactants the repulsion is of an electrostatic character (Rosen and Kunjappu, 2012). After measuring the zeta potential value, it was found that EM has a slightly negative zeta potential value of about −3 mV. Zeta potential measurements of EM indicate that the interface is slightly negatively charged which will enhance the stability of the EM by causing double-layer repulsion between droplets. In addition to surfactant, the origin of interface charge depends on the composition of oil, pH, and electrolytes present in the water phase.

### Antimicrobial activity

All clinical strains were MDR and two of them GN249 and GN321 are invasive, as it was previously shown by Lukovic et al. (2020). The microdilution assay pointed out that both of the tested substances expressed notable antimicrobial activity (Table 3). Obtained MIC concentrations were a little bit higher for EO (0.25–0.5 mg/ml), while for EM they were from 0.125 to 0.25 mg/ml. The most sensitive to EO was the GN249 clinical isolate (MIC 0.25 mg/ml), while the susceptibility of all isolates to EM was the same (MIC 0.125 mg/ml). All tested strains were sensitive to colistin, and used as a positive control.

**Table 3:** Minimal inhibitory concentration values of cinnamon essential oil and cinnamon emulsion for *Acinetobacter baumannii* strains.

Strain	MIC values for EO^*^	MIC values for EM^*^	Colistin^*^
ATCC 19606	0.5	0.25	0.25
GN249	0.25	0.125	0.5
GN321	0.5	0.125	0.5
GN1028	0.5 0.5	0.125	0.25
GN1031	0.5	0.125	0.5

* Concentrations were presented as mg/ml. The experiment was done in triplicates.

### Biofilm assay

#### Antibiofilm activity

**Figure 4:**
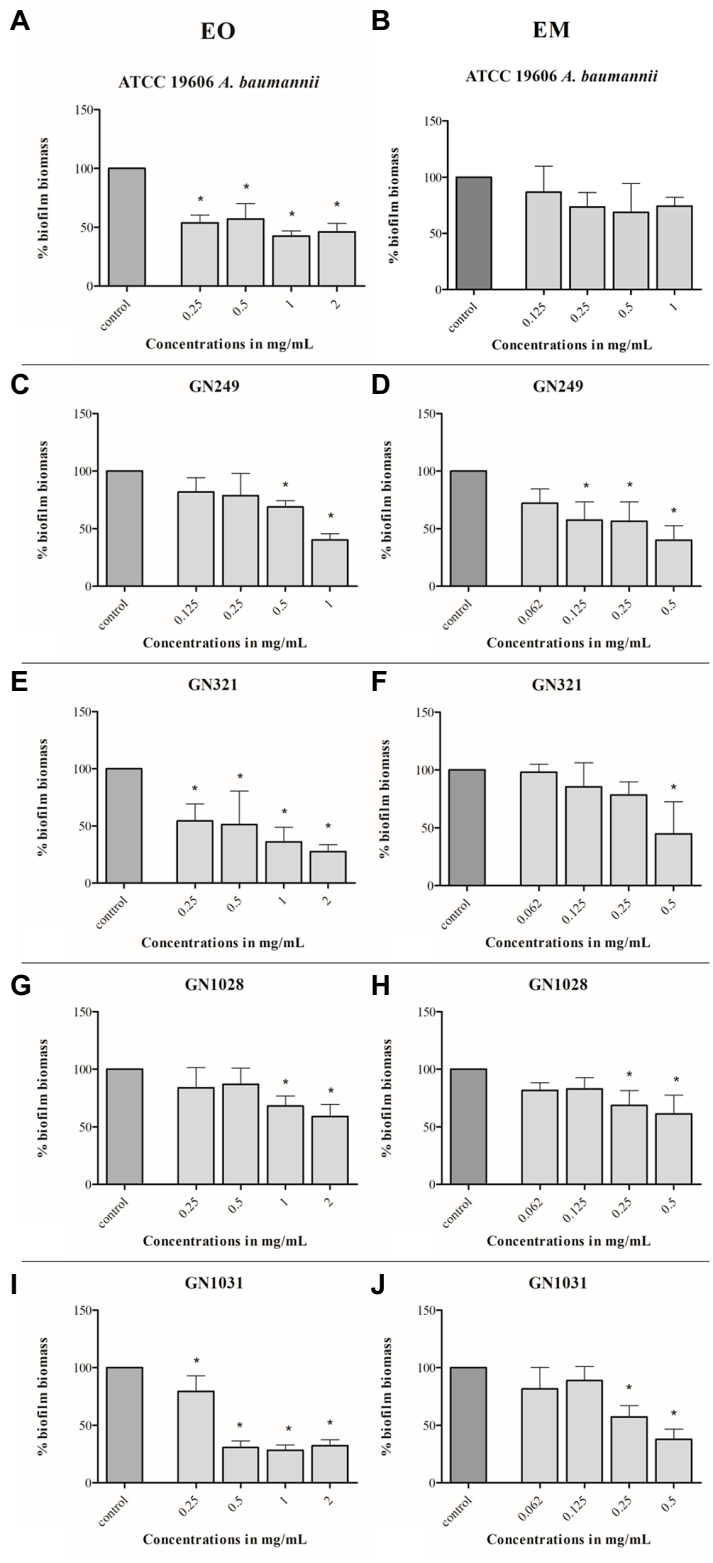
Antibiofilm effects of cinnamon essential oil **(A,C,E,G,I)** and cinnamon emulsion **(B,D,F,H,J)** on *Acinetobacter baumannii* strains. The share of pure cinnamon essential oil in emulsion was 20% *v*/*v*. The range of minimal inhibitory concentration values for cinnamon essential oil were from 0.25 to 0.5 mg/ml and for cinnamon emulsion from 0.125 to 0.25 mg/ml. The experiment was done three times in hexaplicates. Statistical significance was ^*^p < 0.05.

The potential of test substances to prevent biofilm formation, measured by CV staining, is graphically illustrated in Figure 4. The concentrations range used in this assay were defined for each strain according to their MIC values (1/64MIC–MIC). In all tested strains biomass was lower, compared to control. Comparing of overall activities indicated that both dose-dependent (both substances in ATCC 19606, EM in GN249, EO in GN1028) and U-shape (all the rest cases) forms of the response were observed. Further on, comparing the biofilm biomass obtained at the same concentration of the oil (applied as a pure EO or within the EM) showed that emulsification clearly increased the activity against all tested strains except GN1028. The highest decreasement of biofilm biomass was determined in GN1028 and GN321 isolates for EO (81%) and EM (67%), respectively. Following activities are also worth noting: concerning EO inhibitions were 54 and 52% for GN321 and ATCC19606, respectively, while EM inhibited biofilm formation of ATCC19606, GN1031 and GN249 (57, 55 and 47%, respectively).

#### Biofilm dispersal activity

**Figure 5:**
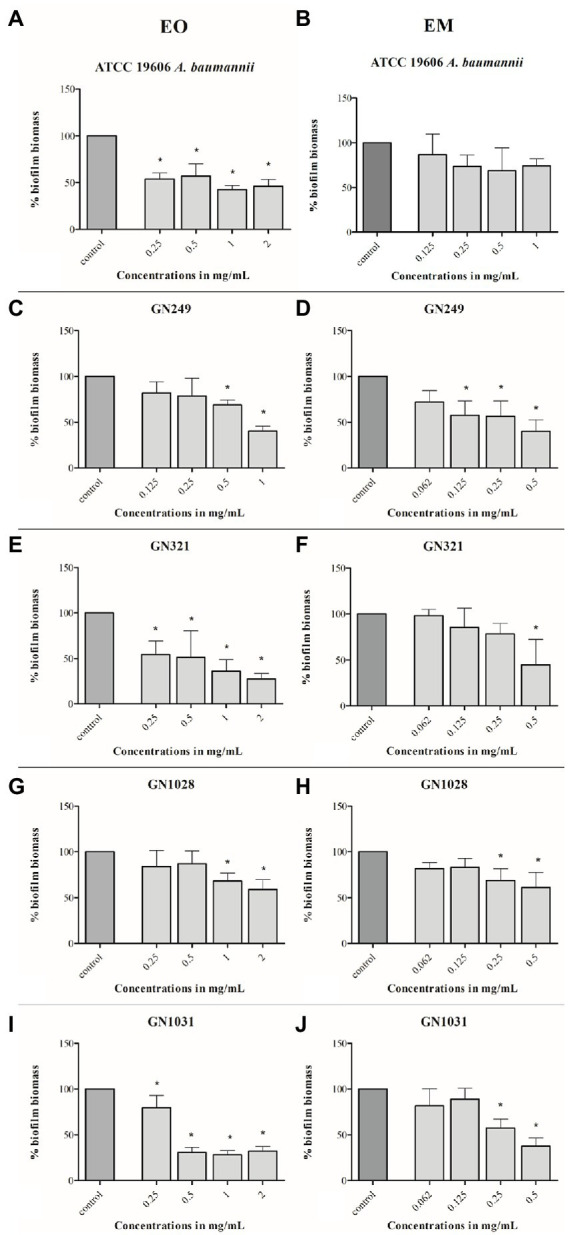
Effects of cinnamon essential oil **(A,C,E,G,I)** and cinnamon emulsion **(B,D,F,H,J)** on already formed biofilm of *Acinetobacter baumannii* strains. The share of pure cinnamon essential oil in emulsion was 20% *v*/*v*. The range of minimal inhibitory concentration values for cinnamon essential oil were from 0.25 to 0.5 mg/ml and for cinnamon emulsion from 0.125 to 0.25 mg/ml. The experiment was done three times in hexaplicates. Statistical significance was ^*^p < 0.05.

As for biofilm inhibition, the applied concentration range for this test is also determined concerning the MIC value (1/2MIC–4MIC). Both, EO and EM showed notable effects according to the percentage of decreased biofilm biomass. All the results were presented graphically in Figure 5. Dose-dependent responses were determined in all tested strains with both tested substances. The most sensitive strain was GN1031, with the highest reduction of pre-formed biofilm biomass valuing 71 and 62% for EO (1 mg/ml) and EM (0.5 mg/ml), respectively. Concerning the highest numerical values of the reduction, it seems that the EO induced higher biofilm dispersal activity, especially in the cases of GN321 and GN1031 isolates. However, if one takes into account the fact that the concentration of EO within emulsion is 5-fold lower than the presented concentrations of whole EM, it can be noted that the emulsification increased oil activity at least for the GN249 strain, which was isolated from central venous catheter. The strongest effect was determined for GN1031 isolate for EO up to 71% and for EM up to 62% of biofilm dispersal.

**Figure 6:**
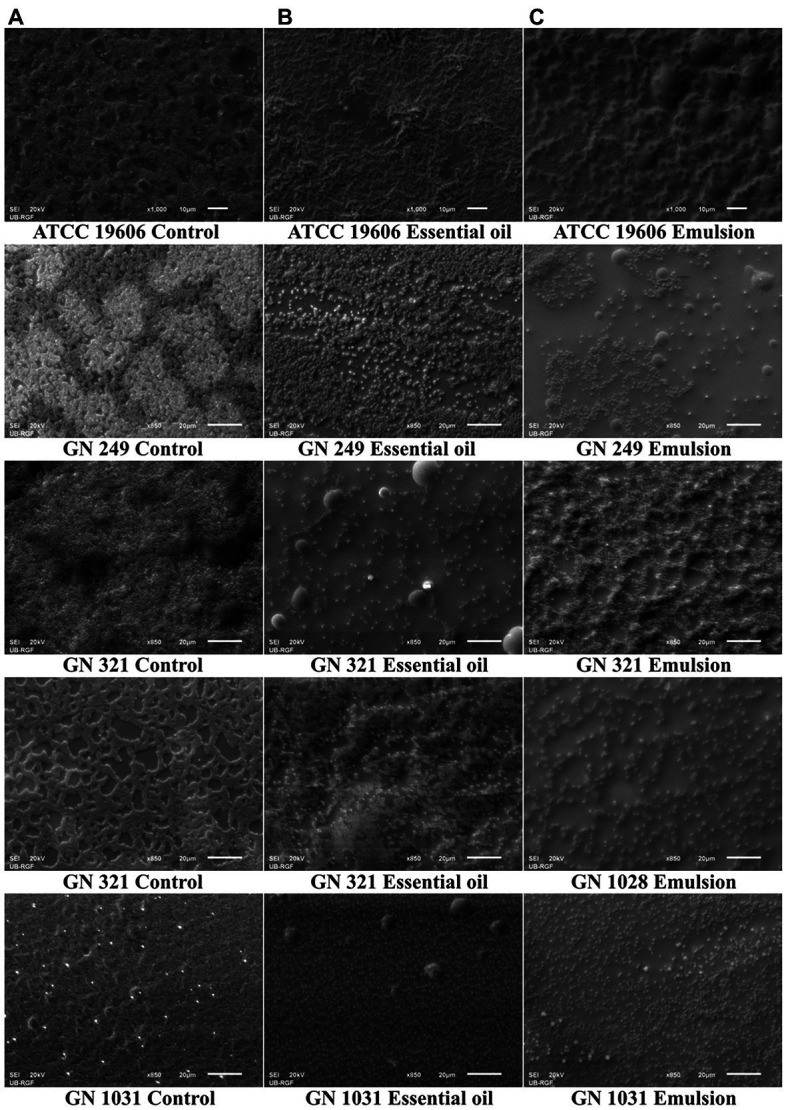
Scanning electron microscopy micrographs of *Acinetobacter baumannii* ATCC 19606 and clinical strains biofilms. On the left side are not treated biofilms used as controls **(A)**, in the middle are biofilms treated with the cinnamon essential oil **(B)**, and on the right side are biofilms treated with the cinnamon emulsion **(C)**. Concentrations used for biofilm treatment with cinnamon essential oil and emulsion was the highest applied concentration used in biofilm dispersal assay. All micrographs are presented with the magnification × 850 or × 1,000, with the scale bars in the down-right corner of the pictures 20 or 10 μm, respectively.

Scanning electron microscopy (SEM) micrographs were analyzed on already formed biofilms *A. baumannii* with the highest tested concentrations of cinnamon essential oil and its emulsion. It is visible that the effect of EO and EM are in accordance with the results obtained in biofilm dispersal assay. In the micrographs it is noticeable the differences between the control and the treated biofilms architecture (Figure 6). It is important to note that in all clinical isolates significant morphological changes was observed in treated biofilms.

### Cytotoxicity assay

**Figure 7:**
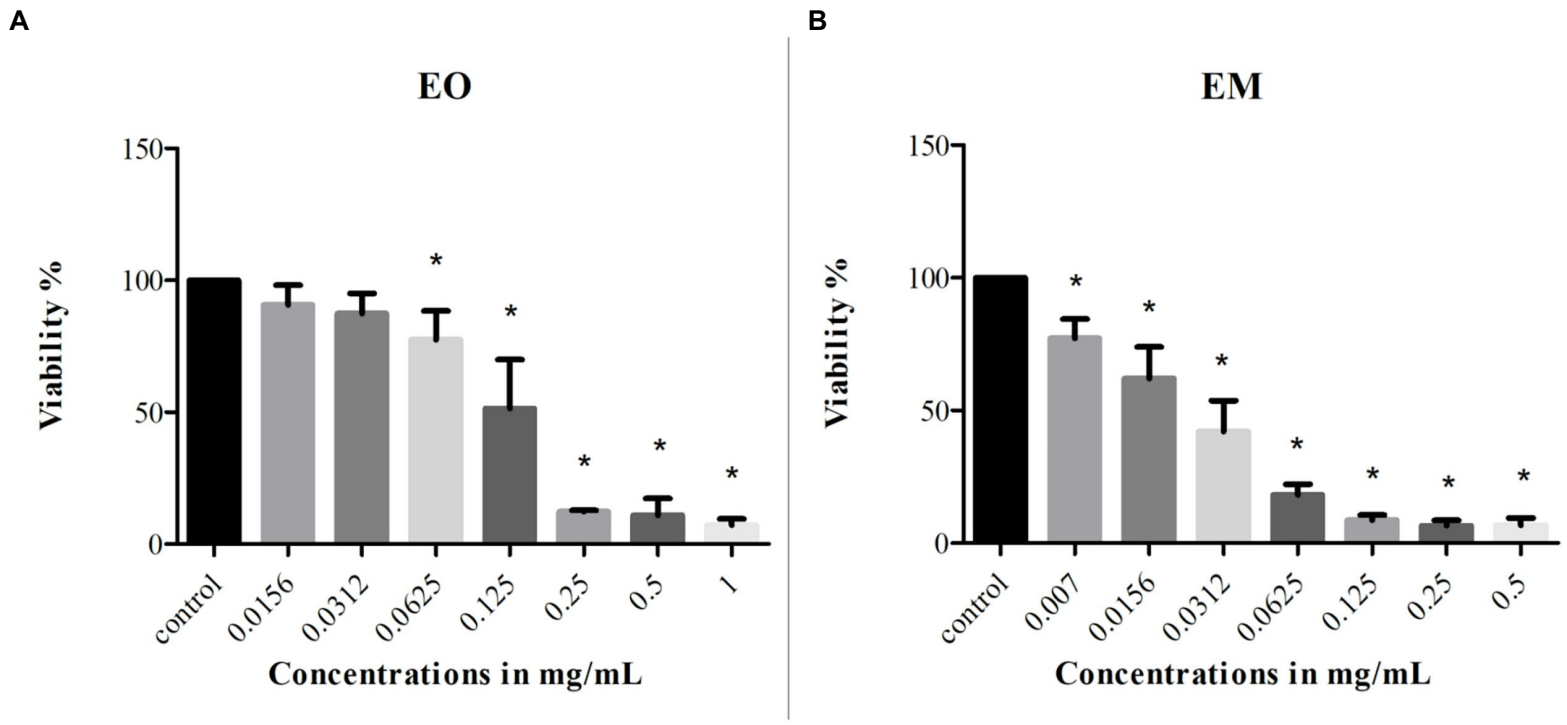
Cytotoxicity of cinnamon essential oil **(A)** and cinnamon emulsion **(B)** on MRC-5 (fetal fibroblasts) cell line. The experiment was done three times in hexaplicates. Statistical significance was ^*^p < 0.05.

MTT assay was used to monitor the cells’ survival after the 24 h treatment with EO and EM. The effect was studied on the MRC-5 cell line and the concentration ranges of test substances were 0.0156^–1^ mg/ml and 0.007–0.5 mg/ml for EO and EM, respectively. The results shown in Figure 7 exhibited that both tested substances inhibited cell growth in a dose-dependent manner. The percentage of cell viability was in a range from 7 to 90% and from 6 to 77% for EO and EM, respectively. The three highest concentrations (0.25–1 mg/ml of EO and 0.125–0.5 mg/ml for EM), exhibited great cytotoxicity where the cell viability was less than 10%.

According to the IC_25_ and IC_50_ values shown in Table 4 it was clear that EM induced higher cytotoxicity. Moreover, if one takes into account that the EO share within EM is 20%, the emulsification process increased the cytotoxic activity of EO many times over.

**Table 4:** Inhibitory concentration values of cinnamon essential oil and its emulsion tested on MRC-5 cell line.

mg/ml	EO	EM
IC_50_	0.13	0.025
IC_25_	0.067	0.007

### Comet assay

**Figure 8:**
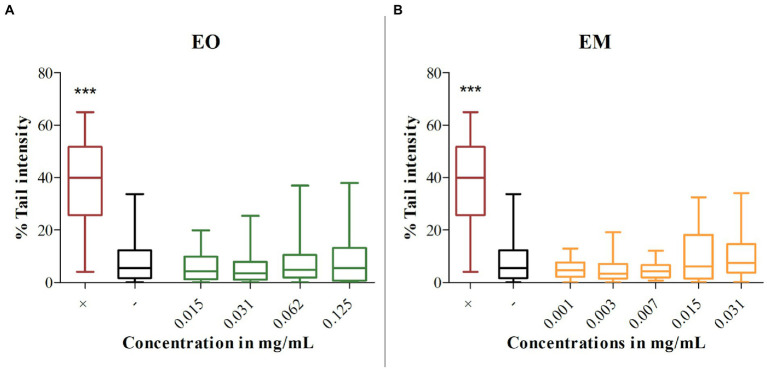
Genotoxicity of cinnamon essential oil **(A)** and cinnamon emulsion **(B)**. Horizontals lines through boxes are medians of the scored nuclei per tested substances. Statistical significance was tested using the Mann–Whitney *U*-test (^***^p < 0.001) concerning control (–). 0.1% H_2_O_2_ was used as a positive control (+).

Using the alkaline comet test genotoxicity was assessed for the chosen concentrations of EO and EM on the MRC-5 cell line. Treatments were compared with the negative control (untreated cells) and as a result none of the tested concentrations showed a statistically significant amount of DNA damage as presented in Figure 8. Although literature data commonly recommend 80% cell viability as a threshold for genotoxicity screening in comet assay (Tice et al., 2000; Žegura and Filipič, 2004), we included additionally the cytotoxic concentrations to determine the secondary genotoxic effect.

## Discussion

In recent years, natural compounds have been at the peak of scientific interest due to their wide spectrum of different secondary metabolites. As the secondary metabolites usually have antimicrobial activity, the idea was to use them to fight against different bacterial infections. This new approach could contribute to solving the problem we face today with antimicrobial resistance (Lelario et al., 2018). Following this idea, in our study we examined cinnamon bark EO. Of overall 38 different identified compounds of cinnamon EO, the most abundant is *trans*-cinnamaldehyde (61.9%), which was expected and is in correlation with other works, where *trans*-cinnamaldehyde was also the most prevalent component in cinnamon bark EO (Yap et al., 2015; Kaskatepe et al., 2016). This is also in accordance with Dussault et al. (2014) who showed that *trans*-cinnamaldehyde is usually most present in both the bark and leaves. According to literature data the similarity of the chemical profile of EOs could be partially ascribed to the plant organ and the cell type where the EOs are synthesized (Figueiredo et al., 2008; Barra, 2009; Prins et al., 2010). The other abundant components of EO are eugenol and cinnamyl acetate, whose content is slightly higher in our study than in Behbahani et al. (2020). Anethole as a component is also more highly presented in this study than usually. Observed differences in the cinnamon oil between the studies could be ascribed to the variability in the process of EO distillation, as well as geographical position and external conditions such as temperature or rainfall, which are recognized as preconditions for the diversity of chemical compositions (Figueiredo et al., 2008). As it is well known, EOs have good potential to be used against different bacterial pathogens. However, in order to be used in medical treatments, it is necessary to solve the problem of their poor solubility (Pavoni et al., 2020). To overcome this issue, we synthesized EM from cinnamon EO.

According to Gupta et al. (2016), the desirable size of particles within nanoemulsions is between 20 and 500 nm, larger and smaller sizes of emulsions are called macro- and micro-emulsion. The point of a particle’s small size is to increase stability and bioavailability by embedding the oil in drops that are surrounded by surfactant molecules. Particle size depends on the amount of surfactant and possible additives in surfactant (Gupta et al., 2016). As in this study, EM had multimodal particle size, this could be ascribed to the aggregation and flocculation of the oil droplets, or even the pH of the whole EM system (Sharma et al., 2022). Nevertheless, according to the literature, the synthesis of EM could improve Eos antibacterial properties (Franklyne et al., 2016).

*Acinetobacter baumannii* is one of the most problematic MDR species, which can be found in hospitals. It is extremely resistant to a broad spectrum of different classes of antibiotics, as it was been shown by our group (Lukovic et al., 2020). Clinical isolates were sensitive only to colistin. Because of the seriousness of the problem with *A. baumannii*, especially in the light of its strong tendency to form biofilms (Upmanyu et al., 2022), treatment alternatives are urgent to be found and better understood. The pre-screening of antibacterial activity of cinnamon EO has shown a notable antimicrobial effect against *A. baumannii* which is in accordance with data from literature (Chouhan et al., 2017; Intorasoot et al., 2017; Aumeeruddy-Elalfi et al., 2018). *Trans*-cinnamaldehyde is one of the most abundant compounds (61.9%) in tested EO, and it has been proven to possess different biological activities among which is an antimicrobial one (Doyle and Stephens, 2019). Furthermore, an increase in its content within Eos resulted in higher antimicrobial activity (Ribeiro-Santos et al., 2017). Therefore, the strong antimicrobial activity of our EO could be attributed to the presence of *trans*-cinnamaldehyde. However, the contribution of other constituents, as well as their possible synergistic interactions cannot be excluded (Bakkali et al., 2008; Huang et al., 2021).

Further, to improve EO activity and increase its bioavailability which is necessary for possible medicinal applications, we synthesized EM. The antimicrobial activity of EM was more pronounced, especially in light of the exact share of EO (20%) within it. We assumed that EM, because of the lower sizes of droplets, more easily penetrated the cells than EO and increased antimicrobial efficiency. The investigations that comparably analyzed the activities of the EOs and their emulsions are very scarce. Similar to our work, Yazgan et al. (2019) compared the antimicrobial effect of lemon EO and its nanoemulsion against food-borne pathogens and spoilage bacteria and exhibited that lemon nanoemulsion possessed a stronger antimicrobial effect.

Infections associated with *A. baumannii* present a serious problem, because of the difficulty of its treatments. When bacteria form a biofilm, doses of antibiotics used against them are very often ineffective (Wolfmeier et al., 2018). From that point of view, searching for potent antibiofilm agents, especially against *A. baumannii* biofilm which has been poorly investigated yet, is of utmost importance. Moreover, results indicating the notable antibacterial potential of both EO and EM in microdilution assay encouraged investigation of their antibiofilm properties. For that reason, in further work, we have screened for both, inhibitory effect on biofilm formation and disruptive potential against pre-formed biofilm.

Concerning antibiofilm activity, literature data pointed out that both cinnamon EO and trans-cinnamaldehyde are embedded with notable potential against different bacterial species. Cinnamaldehyde changes the integrity and permeability of the plasma membrane, but also interferes with quorum sensing, which provides inhibition of biofilm formation (Mohamed et al., 2018). The results obtained in this study showed that EO effectively prevented the formation of biofilm biomass of all tested strains, while the effect of EM was observed on all the strains except GN1028. It is important to note that significantly lower concentrations of EM in comparison to EO were effective, indicating the potential of emulsification to upgrade activity. As it was previously stated, EO and EM in some strains induced a U-shaped response, i.e., the highest antibiofilm activity was observed at medium tested concentrations. This type of response is better known as hormetic, i.e., the agents could induce either beneficial or detrimental effects, depending on applied concentration (Bellavite et al., 2010; Calabrese, 2010). A similar result has been determined previously for emodin, which also induced a U-shape response in both biofilm formation and disruption screening (Đukanović et al., 2022). Taking into account that the antibiofilm activity of cinnamon EO and especially EM, has not yet been fully investigated, the obtained results are significant especially when we consider that inhibition of initial cell binding can prevent further colonization and development of infection, and is therefore proposed as one of the strategies against the formation of bacterial biofilms (Ghosh et al., 2020).

Further, we examined the effect of EO and EM on the dispersal of already formed biofilm. The bacterial biofilm community is exceedingly resistant to the antibiotic and it is a severe challenge to combat *A. baumannii* biofilm infection. Here, we showed that EO strongly influenced dispersal of biofilm of all tested *A. baumannii* strains, while EM dispersed biofilms of clinical strains only, but at higher tested concentrations. Although comparison of percentages of biofilm dispersion indicated that activity of EM was less pronounced than of EO, the fact that EO share within EM is 20% should be taken into consideration. Indeed, the highest activity of NA was observed at a concentration of 0.5 mg/ml, containing only 0.1 ml of EO. In other words, there is an indication that emulsification of EO also increased the eradicative potential of cinnamon oil. Similar to these results, Condò et al. (2020) demonstrated strong dispersal activity of cinnamon extract on already formed biofilm against ATCC strains of *P. mirabilis*, *K. pneumoniae*, *P. aeruginosa* and *E.coli.* The significance of this result is of special interest since literature data concerning the eradication of *A. baumannii* biofilm is scarce (Govindan et al., 2022). Concerning cinnamon EO and its emulsion, this is the first report indicating that they could be used to eradicate *A. baumannii* biofilm.

Bearing in mind that EOs can be toxic due to their chemical profiles and interactions between the components (Aumeeruddy-Elalfi et al., 2018), it is necessary to estimate the safety of their application. To pre-screened in *in vitro* conditions, the risk of use of cinnamon EO and its EM, cytotoxicity and genotoxicity testing were performed on normal fetal lung fibroblasts (MRC-5 cell line). Obtained results showed the existence of a cytotoxic dose–response in the treatment with both substances. The higher tested concentrations for EO and all tested for EM expressed a statistically significant cytotoxic effect. Similar results were obtained for cinnamon EO on normal rat fibroblasts (cell line F2408), where nontoxic concentration was approximatively 10 μg/ml, while the higher ones were cytotoxic (Unlu et al., 2010). A higher level of EM induced cytotoxicity could be due to the small size of particles and their easier passage into the cells (Weerapol et al., 2022). Although both EO and EM showed a cytotoxic effect, that does not exclude them from further research which should be performed to minimize cytotoxicity and enable their possible use. Moreover, the non-cytotoxic EO concentrations exhibited the notable potential to inhibit biofilm formation (up to 0.031 mg/ml). On the other hand, although EM was cytotoxic in all applied concentrations, one should bear in mind that further EM upgrading could be used to minimize the harmful effect and enable the utilization of valuable properties, i.e., an antibiofilm effect. Further on, the obtained results of the comet assay are very promising due to the fact that none of the tested concentrations provoke DNA damage. It was already proved that cinnamic acid as one of the components which can be found in cinnamon EO does not have a genotoxic effect (Maistro et al., 2011). This study’s results are very similar with the work of Kozics et al. (2019). They tested genotoxicity of the *Cinnamomum cassia* EO and none of the tested concentrations were genotoxic.

In conclusion, cinnamon EO exhibited notable antibacterial and antibiofilm activities, i.e., preventing biofilm formation and successfully eradicating pre-formed biofilms of tested *A. baumannii* strains. The process of emulsification additionally increased both antibacterial and antibiofilm effects. However, the high cytotoxicity of EM indicates that further investigation and upgrading of emulsion is needed, to preserve antibiofilm effect and to diminish cytotoxic properties. Nevertheless, the obtained results are encouraging and indicate that both EO and especially its emulsion with improved bioavailability have the potential to be used in therapy against *A. baumanni* infections. This approach could be considered a significant shift in the fight against this emerged nosocomial pathogen and its antimicrobial resistance.

## Data availability statement

The original contributions presented in the study are included in the article/supplementary material, further inquiries can be directed to the corresponding author.

## Author contributions

TG and DM-Ć contributed to the conception and design of the study and wrote the first draft of the manuscript. SV contributed to the conception and design of the study. BN wrote sections of the manuscript and contributed to the experimental design. MK contributed to the synthesis of emulsion. MS contributed to the synthesis, characterized emulsion, and wrote the section of the manuscript. SĐ contributed with the chemical analysis. SC contributed with statistical analysis. All authors contributed to the article and approved the submitted version.

## Funding

This work was supported by the Ministry of Education, Science and Technological Development of Republic of Serbia (451–03-68/2022–14/200178 and 451–03-68/2022–14/200175).

## Conflict of interest

The authors declare that the research was conducted in the absence of any commercial or financial relationships that could be construed as a potential conflict of interest.

## Publisher’s note

All claims expressed in this article are solely those of the authors and do not necessarily represent those of their affiliated organizations, or those of the publisher, the editors and the reviewers. Any product that may be evaluated in this article, or claim that may be made by its manufacturer, is not guaranteed or endorsed by the publisher.
